# A Facile Method for Fabricating a Monolithic Mullite Fiber-Reinforced Alumina Aerogel with Excellent Mechanical and Thermal Properties

**DOI:** 10.3390/gels8060380

**Published:** 2022-06-15

**Authors:** Lin Liu, Xiaodong Wang, Ze Zhang, Yixin Shi, Yicheng Zhao, Shiqi Shen, Xiandong Yao, Jun Shen

**Affiliations:** 1 Shanghai Key Laboratory of Special Artificial Microstructure Materials and Technology, School of Physics Science and Engineering, Tongji University, Shanghai 200092, China; 1930976@tongji.edu.cn (L.L.); zzzhangze@163.com (Z.Z.); 2 College of Chemistry and Chemical Engineering, Shaoxing University, Shaoxing 312000, China; syx19817757085@163.com (Y.S.); zyc010621@163.com (Y.Z.); m15968983815@163.com (S.S.); nanoyxd@163.com (X.Y.)

**Keywords:** alumina aerogel, mullite fiber, sol-gel method, mechanical properties, thermal properties

## Abstract

Alumina aerogels are considered to have good application prospects in the high-temperature field. In this study, monolithic mullite fiber-reinforced alumina aerogels with excellent mechanical and thermal properties were synthesized via a facile method without the use of any chelating agents. This method successfully avoids the introduction of impurities during the use of catalysts and chelating agents while greatly reducing gelation time, and thus helps mullite fibers to uniformly disperse in the sol. The compressive stress at 80% strain of the obtained mullite fiber-reinforced alumina aerogels was as high as 16.04 MPa—426% higher than that of the alumina aerogel without the addition of mullite fibers. Regarding thermal properties, the shrinkage of the mullite fiber-reinforced alumina aerogels (AM) samples was less than 1% after heat treatment at 1300 °C for 2 h. Furthermore, the rear-surface temperature of the AM samples burned by a butane blow torch was only 68 °C. These outstanding properties make AM samples promising for application in thermal insulation materials in high-temperature fields such as aerospace and industrial thermal protection in the future.

## 1. Introduction

Aerogels are a new type of nano-porous material that have a wide range of applications such as catalysis, thermal insulation and energy due to their low density, high porosity, low thermal conductivity and other superior characteristics [1,2,3,4,5,6]. Compared with silica aerogels, alumina aerogels have better thermal stability and can still maintain high structural integrity and high porosity at high temperatures (>800 °C) [7,8,9]. Therefore, alumina aerogels are considered to have good application prospects in high-temperature fields such as thermal insulation for aerospace and industrial thermal protection [7,8,10,11]. Alumina aerogels are usually prepared by the sol-gel method and supercritical fluid drying [10,12]. Inorganic aluminum salts [12,13,14] and organic aluminum alkoxides [10,15,16] are generally selected as precursors for the preparation of alumina aerogels. Aerogels prepared from organic aluminum alkoxides generally exhibit better high-temperature-resistant performance [7,8,17]. Zu et al. [10] prepared monolithic Al_2_O_3_ aerogels with a high specific surface area using aluminum sec-butoxide as the precursor and nitric acid as the catalyst. In this method, no chelating agents were used during the synthesis process. The Epoxide addition method is a simpler method for preparing alumina aerogels with inorganic aluminum salts. Baumann et al. [12] prepared Al_2_O_3_ aerogels with a low-density and high specific surface area by carbon dioxide supercritical drying, using AlCl_3_·6H_2_O and Al (NO_3_)_3_·9H_2_O as precursors and propylene oxide as the gel accelerator. However, these alumina aerogels are always easily cracked due to their inherently fragile skeleton and high porosity, which greatly limits their performance and application [8,10,18,19,20,21]. To improve the mechanical properties of alumina aerogels, fibers and whiskers [22,23,24,25,26,27] have been introduced into the skeleton of alumina aerogels to reinforce the fragile skeleton [28,29]. For example, introducing surface-modified quartz fibers into alumina aerogels can significantly improve the compressive strength of the final samples [30]. Julien et al. [31] prepared alumina aerogels with high mechanical properties by atmospheric drying and carbon dioxide supercritical drying using short manmade cellulose fibers as reinforcing materials. The effects of fiber length and volume percentage on the properties of composite aerogels have also been investigated.

Mullite fibers [32,33] have the advantages of high-temperature resistance, low density, low thermal conductivity and good chemical stability compared to other reinforcing materials. Moreover, mullite fibers have good mechanical properties. Combining their various advantages, mullite fibers are an effective reinforcement for alumina aerogels. Over the past few years, mullite fibers have been processed into specific sizes and shapes to suit different applications [25]. Mullite fiber felts with specific sizes and densities have been directly dipped into alumina sols to produce large-scale aerogel composites [27]. In addition, different infrared opacifiers such as carbide [22,34] and titanium dioxide [35] have also been added to mullite fiber-reinforced materials to better reduce high-temperature radiation. Furthermore, growing mullite whiskers on the surface of mullite fibers can significantly enhance the interface between fibers and aerogels, which greatly improves the mechanical properties of mullite fiber–aerogel composites [24]. In these studies, the materials used were all mullite fiber felts or specially shaped reinforcements made of mullite fibers. The reported preparation processes were complicated, including the preparation of mullite fiber reinforcements, the in-situ growth of whiskers on mullite fibers and the addition of chelating agents. These problems greatly increase the cost of preparation, which is not beneficial to its production. Besides this, the weight percentage of mullite fiber felts in the composite sample was relatively high.

Here, a facile method was developed to prepare mullite fiber-reinforced alumina aerogels (AM) without using any chelating agent. Short mullite fibers of 5 to 10 microns in diameter and 100–200 microns in length were used as reinforcements. The short mullite fibers did not need to be manufactured into fiber felts, which reduced the preparation cost and time. Both AlCl_3_·6H_2_O and aluminum sec-butoxide (ASB) were used as precursors. ASB was partially hydrolyzed to form Al(OH)_n_(OC_4_H_9_)_3−n_ after hydrolysis, while AlCl_3_·6H_2_O was also hydrolyzed in solution to form hydrogen ions and hydroxylated complexes [Al(OH)_h_(OH_2_)_6−h_]^(3−h)+^. The formed hydrogen ions provided an acidic condition to accelerate the hydrolysis of the ASB. After mixing with the partially hydrolyzed ASB, the condensation reactions between the Al–OH or Al–OC_4_H_9_ from the Al(OH)_n_(OC_4_H_9_)_3−n_ and the Al–OH from the [Al(OH)_h_(OH_2_)_6−h_]^(3−h)+^ proceeded, and finally, formed an alumina gel with a cross-linked chain structure. Monolithic mullite fiber-reinforced aerogels could be obtained after ethanol supercritical drying. By adjusting the content of the mullite fibers from 0% to 25%, the effects of the mullite content on the mechanical and thermal properties of the obtained samples were studied.

## 2. Results and Discussion

**Figure 1:**
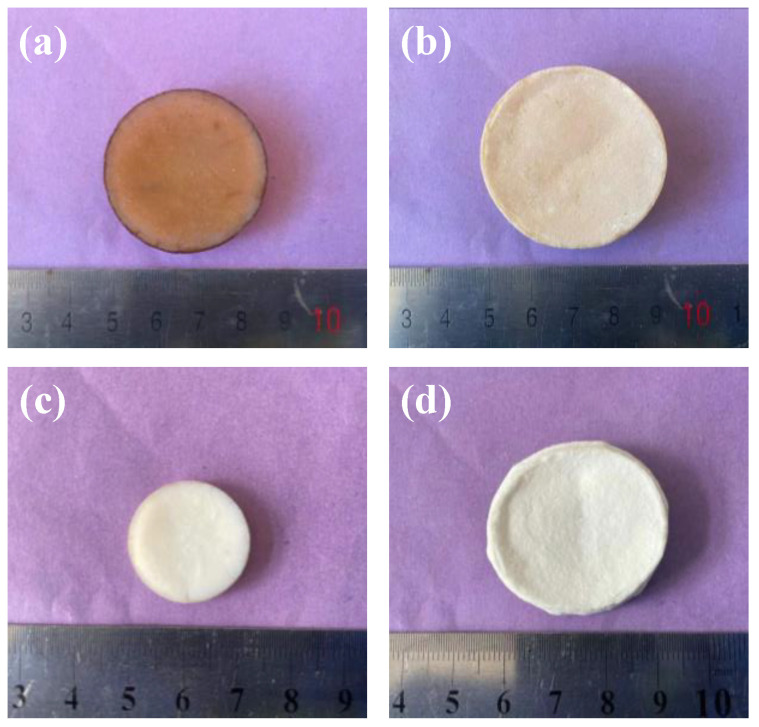
Photographs of AM0 (**a**), AM4 (**b**) as prepared and AM0 (**c**), AM4 (**d**) after heat treatment at 1300 °C.

The samples, with different mullite fiber contents of 0 wt.%, 5 wt.%, 10 wt.%, 15 wt.%, 20 wt.% and 25 wt.%, were named AM0, AM1, AM2, AM3, AM4 and AM5, respectively. The photograph of the as-prepared AM0 and AM4 samples and the same samples after heat treatment at 1300 °C are shown in Figure 1. As shown in Figure 1a,b, the as-prepared AM0 and AM4 samples showed a brownish-yellow color due to the decomposition of the organic groups inside the sample; the shrinkage of the AM4 was smaller than that of the AM0. After heat treatment at 1300 °C, both samples AM0 and AM4 exhibited a white color (see Figure 1c,d, respectively). Besides this, the AM0 sample exhibited large shrinkage, while the shrinkage of the AM4 sample was relatively small. These results indicate that the introduction of mullite fibers can significantly improve the high-temperature resistance of alumina aerogels.

**Figure 2:**
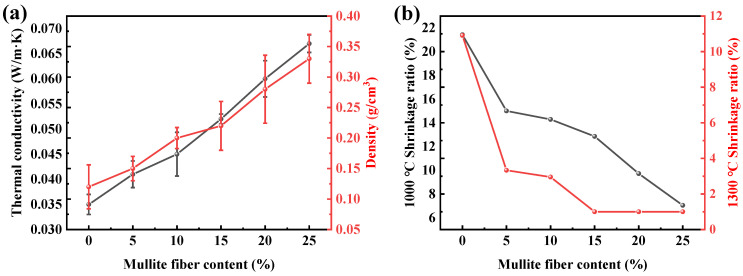
Density and thermal conductivity of AM samples as a function of mullite fiber content (**a**); thermal shrinkage ratio of AM samples after heat treatment at 1000 °C and 1300 °C as a function of mullite fiber content (**b**).

The density and room temperature thermal conductivity curves of the AM samples are shown in Figure 2a. The density of the samples increases from 0.12 to 0.33 g/cm^3^ with increases in the mullite fiber content, which leads to increases in the room temperature thermal conductivity (from 0.032 to 0.050 W/(m·K)). The total effective thermal conductivity of the aerogel material is composed of the solid thermal conductivity, gaseous thermal conductivity and radiative conductivity [36,37] as follows:

$$\lambda_{total}=\lambda_{s}+\lambda_{g}+\lambda_{r}=\frac{1}{3}\int C_{v}\rho V_{p}l_{p}+\frac{\lambda_{g,0}}{1+\alpha l_{mfp}/l_{cl}}+\frac{16}{3}\frac{\sigma n^{2}T^{3}}{\rho e}$$

where *C_v_* is the specific heat capacity at a constant volume, *ρ* is the density of the porous material, *V_p_* is the mean velocity of the phonon, *l_p_* is the phonon mean free path, *λ_g_*_,0_ is the thermal conductivity in free air, *α* is a constant specific to the gas in the pores, *l_mfp_* is the mean free path of a gas molecule, *l_cl_* is the average pore size in porous materials, *σ* is the Stefan–Boltzmann constant, *n* is the effective index of refraction, *T* is the absolute temperature and *e* is the effective specific extinction coefficient. At room temperature, the value of the radiant heat conduction is almost negligible. Therefore, the thermal conductivity of the AM samples at room temperature is mainly determined by the solid thermal conductivity and gaseous thermal conductivity. The solid thermal conductivity of aerogels is closely related to the density and the average velocity of the phonons in the aerogel skeleton. The average velocity of the phonons is positively related to the contact degree of the nanoparticles in the aerogel skeleton. As the mullite fiber content increases, the solid content of the aerogel increases, which leads to an increase in the density and the solid thermal conductivity. Furthermore, aerogel has abundant nano-cavities, and its pore diameter is smaller than the mean free path of air molecules, which effectively reduces gas convection and collision heat transfer between gas molecules and thus has extremely low gaseous thermal conductivity. With increases in the mullite fiber content, a part of the nano-cavities inside the sample is filled with mullite fibers, which reduces the gaseous thermal conductivity of the sample. With increases in the mullite fiber content, the increase in the solid thermal conductivity is larger than the decrease in gaseous thermal conductivity, which results in the increase of the total effective thermal conductivity.

Figure 2b presents the thermal shrinkage ratios of samples with different mullite fiber contents after heat treatment at 1000 °C and 1300 °C for 2 h. The shrinkage ratios of the AM samples after heat treatment at 1000 °C tends to decrease with increases in the mullite fiber content, and the shrinkage ratios of the AM samples with different mullite fiber contents (0%, 5%, 10%, 15%, 20%, 25%) are 21%, 15.00%, 14%, 13%, 10% and 7%, respectively. The shrinkage ratios of the AM samples after heat treatment at 1300 °C tends to decrease with increases in the mullite fiber content, and the sample with a 5% mullite fiber content shows a significant reduction in shrinkage compared to the sample without mullite fiber added. This result indicates that the addition of mullite fibers can significantly enhance the temperature resistance of alumina aerogels at 1300 °C. When the mullite fiber content is larger than 15%, the shrinkage ratio of the AM sample is less than 1%. The shrinkage ratios of the AM samples with different mullite fiber contents (0%, 5%, 10%) are 11%, 4% and 3%, respectively.

**Figure 3:**
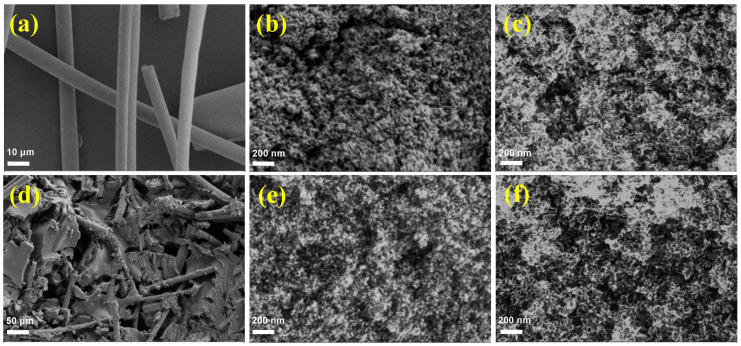
SEM images of the mullite fibers (**a**), AM0 as prepared (**b**) and after heat treatment at 1300 °C (**c**), AM4 as prepared (**d**,**e**), and after heat treatment at 1300 °C (**f**).

Scanning electron microscopy (SEM) images of the AM samples are shown in Figure 3. As shown in Figure 3a, the diameter of the mullite fibers was about 10 μm, and there was little contact between the disorderly arranged fibers. Figure 3d presents an SEM image of the AM4 sample. Mullite fibers were uniformly dispersed in the alumina aerogel skeleton with little contact with each other, and the alumina aerogel particles were closely connected to the mullite fibers. The agglomeration of mullite fibers can contribute to reductions in the thermal properties of aerogels, so the uniform dispersion of mullite fibers in alumina aerogels is crucial. The AM samples prepared by this method can greatly reduce the sedimentation and aggregation of mullite fibers, which is of great help to the improvement of the thermal properties of the AM samples. Figure 3b,e displays SEM images of the AM0 and AM4 samples without any treatment. The alumina aerogels exhibited a rich three-dimensional porous network structure composed of inter-connected particles. Figure 3c,f presents SEM images of the AM0 and AM4 samples after being heated to 1300 °C. The alumina aerogels retained a nano-porous structure, but the nanoparticles in the alumina aerogels transformed into sheet-like structures.

**Figure 4:**
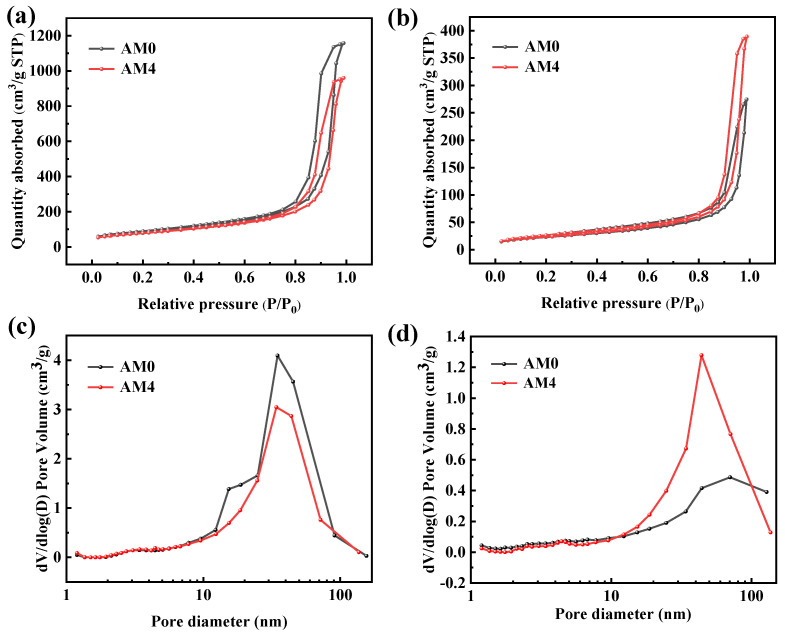
Nitrogen adsorption−desorption isotherms of AM0 and AM4 before (**a**) and after heat treatment at 1300 °C (**b**); Pore size distribution curves of AM0 and AM4 before (**c**) and after heat treatment at 1300 °C (**d**).

Figure 4 presents the N_2_ adsorption/desorption isotherms and pore size distribution of the AM0 and AM4. According to the classification of the IUPAC, the isothermal adsorption/desorption curves of all the samples corresponded to type IV with an obvious hysteresis loop, which indicates a typical mesoporous skeleton structure [38]. However, the type of hysteresis loop was different to the AM samples treated at different temperatures. Figure 4a shows a type H1 hysteresis loop, which indicates a mesoporous structure formed by the accumulation of spherical particles, while Figure 4b shows a type H3 hysteresis loop that demonstrates a slit-shaped mesoporous structure stacked by plate-like particles. These results indicate that heat treatment will change the shape of particles. The pore size distribution curves in Figure 4c show that the pore size of the as-prepared AM0 and AM4 samples were mainly in the range of 10–60 nm; they were smaller than the mean free path of gas molecules, which can prevent air molecules from colliding with each other in the pores. This type of mesoporous structure can greatly reduce gaseous heat conduction. After heat treatment at 1300 °C, as shown in Figure 4d, the pore size distribution curve of the AM0 moves towards the direction of a large hole in the range of 50–100 nm and the pore size distribution peak is 70 nm—which indicates that heat treatment destroys the mesoporous structure and thus generates pores with a larger size. The pore size in sample AM4 is mainly distributed over the range of 20–80 nm, with a distribution peak at approximately 30 nm. The specific surface areas of the AM samples are listed in Table 1. The as-prepared AM0 sample exhibited the largest specific surface area among all the samples. With increases in the mullite fiber content, the specific surface area of the AM samples decreased gradually, from 423 m^2^/g in AM0 to 261 m^2^/g in AM5. After heat treatment at 1300 °C for 2 h, the specific surface area of the AM0 decreased rapidly due to the thermal shrinkage and crystalline transition of the alumina aerogel. However, the mullite fiber-reinforced samples (AM1–AM5) displayed higher specific surface areas than the AM0 after heating at 1300 °C, which probably benefited from the high-temperature thermal stability of the mullite fibers. These results suggest that the addition of mullite fibers can improve the high-temperature thermal stability of alumina aerogels.

**Figure 5:**
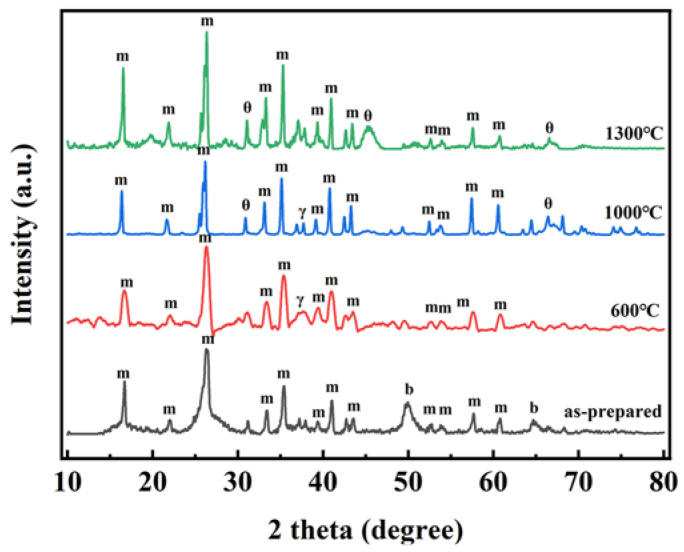
XRD patterns of sample AM4 treated at different temperatures. m: mullite, b: boehmite, γ: γ-Al_2_O_3_, θ: θ-Al_2_O_3_.

The powder X-ray diffraction (XRD) patterns of sample AM4 after being heated at different temperatures are shown in Figure 5. Clear and sharp diffraction peaks in the mullite phase can be observed due to the addition of mullite fibers. The broad diffraction peaks at 49.9° and 64.7° of the as-prepared AM4 were attributed to boehmite (200) and (002) crystal planes (PDF no.21-1307). Boehmite belongs to the orthorhombic system and is a kind of hydrated alumina with incomplete crystallization [4,39,40]. After being heated at 600 °C, the boehmite peaks disappeared and a new diffraction peak at 37.5° was observed, corresponding to the (311) crystal planes of γ-Al_2_O_3_ (PDF no.10-0425). After heating at 1000 °C, broad diffraction peaks at 32.2° and 66.8° were observed, corresponding to θ-Al_2_O_3_ (004) and (215) crystal planes (PDF no.04-0877). γ-Al_2_O_3_ and θ-Al_2_O_3_ peaks both existed in the XRD patterns. After being heated at 1300 °C, the γ-Al_2_O_3_ peak disappeared and the θ-Al_2_O_3_ still existed (an α-Al_2_O_3_ phase was not observed), indicating the excellent thermal stability of AM4. 

**Figure 6:**
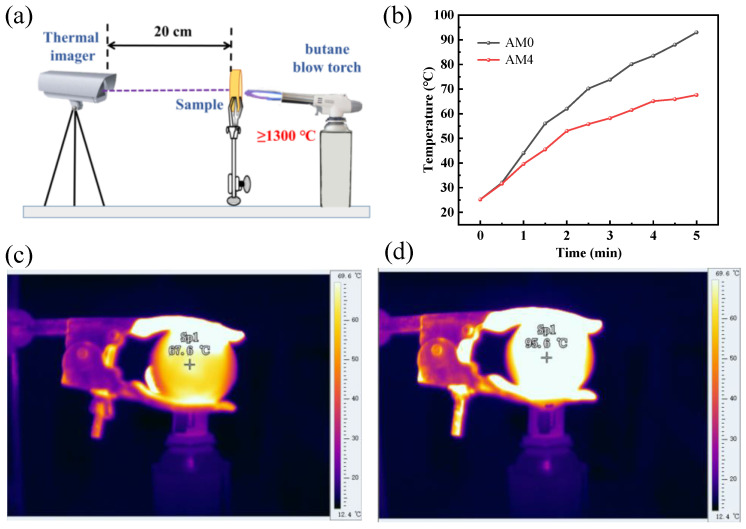
Schematic diagram of the high-temperature thermal insulation test (**a**); rear-surface temperature of AM0 and AM4 as a function of the burning time (**b**); pseudo-color thermal images of the samples after burning for 5 min: (**c**) AM4, (**d**) AM0.

To better evaluate the high-temperature thermal insulation performance of the AM samples, a 1300 °C butane blow torch was used to directly fire one side of the obtained AM samples and the temperature changes of the rear surface of the samples were recorded by an infrared thermal imaging camera. The high-temperature thermal insulation performance of AM0 and AM4 is shown in Figure 6. As schematically shown in Figure 6a, one side of the samples AM0 and AM4 (6 mm thickness) was burned by a butane blow torch, while the temperature of the other side was monitored by an infrared thermal imaging camera. The temperature changes of rear surface of the AM0 and AM4 are shown in Figure 6b. For the AM0 sample, the rear-surface temperature increased to 96 °C within 5 min and exhibited a continuous upward trend. For the AM4 sample, the rear-surface temperature increased to 68 °C only within 5 min and tended to maintain a relatively stable temperature range. The photos of the rear-surface temperature after 5 min of AM0 and AM4 monitored by an infrared thermal imaging camera are shown in Figure 6c,d, respectively. Due to the unique inter-penetrating chain structure and physical properties of alumina, the alumina aerogel showed great thermal insulation properties. Therefore, both AM0 and AM4 showed a low rear-surface temperature increase after being burned for 5 min. Differently, due to its thermal insulation properties and the excellent high-temperature stability of the mullite fibers, the AM4 sample showed a slower temperature rise rate and smaller temperature change compared to the AM0. Such high-temperature thermal insulation performance was also much better compared to the reported results from the literature, e.g., 93 °C in Wang [9] (8 mm thickness, 1300 °C for 5 min), 75 °C in Zu [7] (8 mm thickness, 1300 °C for 5 min) and >200 °C in Ren [41] (10.3 mm thickness, 1200 °C for 5 min).

**Figure 7:**
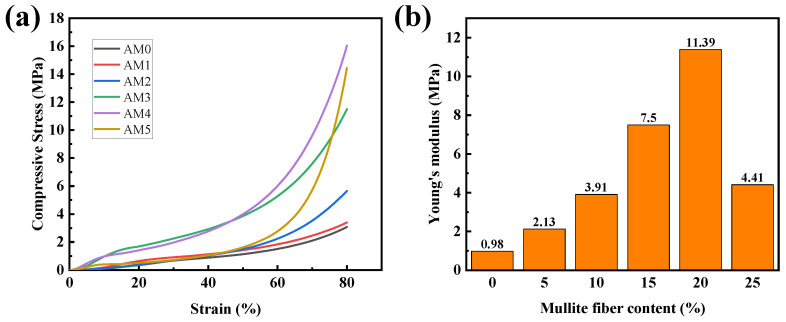
Stress-strain curves (**a**) and Young’s moduli (**b**) of the AM samples.

The mechanical properties of the obtained samples are shown in Figure 7. The stress-strain curves of the AM composites showed no fractures even at 80% strain; the compression process can be divided into three stages—namely the linear stage, yielding stage and densification stage. At the linear stage (0–10% strain), the compressive stress increases almost linearly with increases in compressive strain; the nano-porous alumina aerogel acts as the main load-bearing part. At the yielding stage (10–40% strain), the nano-porous structure of the alumina aerogel is gradually destroyed so that it cannot perform as the main load-bearing part, and the mullite fiber skeleton becomes the main load-bearing body. Therefore, the stress increases at quite a lower rate with the strain compared to the linear stage. At the densification stage (40–80% strain), the mullite fiber skeleton is destroyed, and the AM samples are compacted and become denser. Therefore, the slope of the stress-strain curve increases greatly. The compressive stress of the AM4 (80% strain) was able to reach 16.04 MPa, while the compressive stress of the AM0 was only 3.05 MPa at 80% strain. As we can infer from Figure 7b, the mechanical properties of the AM samples were greatly enhanced due to the addition of mullite fibers. The Young’s modulus of the AM4 was able to reach 11.39 MPa, while the Young’s modulus of the AM0 was only 0.98 MPa. These results demonstrate that the addition of mullite short fibers can greatly enhance the mechanical properties of alumina aerogels, which is greatly beneficial to the application of alumina aerogels.

## 3. Conclusions

In this work, we prepared mullite fiber-reinforced alumina aerogels via ethanol supercritical drying. Both AlCl_3_·6H_2_O and aluminum sec-butoxide were used as precursors, while mullite fibers of 5–10 microns in diameter were used as reinforcement materials. The monolithic alumina aerogel AM4 showed shrinkage of less than 1% after heat treatment at 1300 °C for 2 h. Additionally, the AM4 sample possessed a large number of mesopores, while the AM0 sample had few mesopores after heat treatment at 1300 °C for 2 h. No α-Al_2_O_3_ phase was observed in the XRD pattern of the AM4 sample after heat treatment at 1300 °C for 2 h, which indicates its excellent thermal stability. Meanwhile, the rear-surface temperature of the mullite fiber-reinforced alumina aerogel AM4 only increased to 68 °C after 5 min burning by a butane blow torch. The compressive stress at 80% strain of the AM4 sample reached 16.04 MPa, which was 426% higher than that of the AM0.

## 4. Materials and Methods

### 4.1. Materials

Ethanol (EtOH, 99%) and distilled water, AlCl_3_·6H_2_O (99%) were purchased from the Sinopharm Chemical Reagent Corporation (China). Aluminum sec-butoxide (97%) was obtained from the Zhejiang Ultrafine Powders & Chemicals Corporation (Zhejiang, China). Mullite fibers were purchased from Lei Jing Refractories Co., Ltd. (Zhejiang, China). 

### 4.2. Preparation

**Figure 8:**
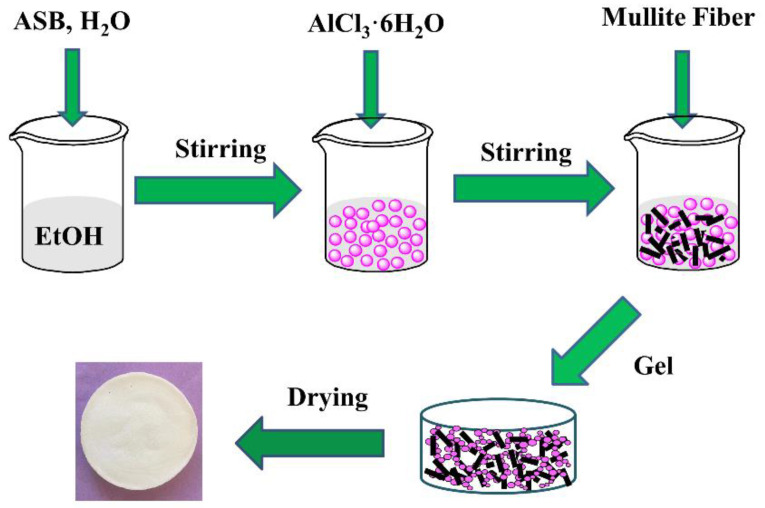
Schematic illustration of the preparation process of the mullite fiber-reinforced alumina aerogels.

As shown in Figure 8, the preparation of mullite fiber-reinforced alumina aerogels can be divided into the following steps:

Firstly, the mixture of ethanol and deionized water was heated to 70 °C. ASB was then dissolved into the mixed solution with vigorous stirring. After becoming stable, the mixture was cooled to room temperature. Secondly, AlCl_3_∙6H_2_O was dissolved in ethanol followed by mixing with the solution above to form alumina sol. The mol ratio of ASB:EtOH:H_2_O and AlCl_3_∙6H_2_O:EtOH were kept at 1:17:0.6 and 1:36, respectively. When the molar ratio of ASB and AlCl_3_∙6H_2_O was 1:0.125, the gel had the best performance. Then, different amounts of mullite fiber (0, 5, 10, 15, 20, 25 wt.%) were added into the alumina sol. This method can avoid introducing impurities during the use of catalysts and chelating agents, while greatly reducing the gelation time. After stirring for about 10 min, the viscosity coefficient of the sol increased significantly, which caused uniformly dispersed mullite fibers in the sol. Gelation took place in 5 min. Since no impurities are introduced in the preparation process, the time for solvent replacement is greatly reduced. The obtained wet gels were further aged in ethanol for 3 days. Lastly, the gels were dried by supercritical ethanol drying. The autoclave was heated to 265 °C at a rate of 1 °C /min, while the pressure of the autoclave reached 12 MPa. This state was kept for 1 h. Then, the ethanol fluid was discharged from the autoclave at a rate of 25 to 30 kPa/min. The AM samples were obtained after the autoclave was cooled to room temperature.

### 4.3. Characterization

The surface morphology of the AM samples was characterized by SEM (XL30FEG, Amsterdam, The Netherlands). The crystal structure of the AM samples was analyzed by a Rigata/max-C diffractometer using Cu-Kα radiation (Rigaku Ultima IV, Tokyo, Japan). The specific surface area and pore-size distribution were determined by the Brunauer–Emmett–Teller (BET) and Barrett–Joyner–Halenda (BJH) methods (Autosorb IQ, Quantachrome Instrument, Boynton Beach, FL, USA) through nitrogen adsorption/desorption characterization. The thermal conductivity was measured by a thermal constant analyzer (Hot Disk TPS2500, Uppsala, Sweden). An infrared thermal imager (FOTRIC 626-L28, Shanghai, China) was used to record pseudo-color thermal images during the high-temperature resistance test. The mechanical properties of the samples were characterized by an electrical material tester (AGS–X, Shimadzu, Suzhou, China).

## Figures and Tables

**Table 1:** Specific surface area of samples as prepared and after heat treatment at 1300 °C.

Sample	Specific Surface Area (m^2^/g)	
	As Prepared	1300 °C
AM0	423	96
AM1	407	117
AM2	374	137
AM3	362	145
AM4	342	112
AM5	261	99

## Data Availability

The data presented in this study are available upon request from the corresponding author.
